# Surgical site infection prevention among surgical healthcare workers in Syria: a nationwide cross-sectional study

**DOI:** 10.1186/s13756-026-01783-y

**Published:** 2026-07-03

**Authors:** Mais Alreem Mohaisen, Mohammad Atia, Bushra Mohammad Motaz Al-Noufi, Bshr Ibrahim Suliman, Rahaf Mohammad Kheir Aljalam, Raghad Darwish Ghilan, Abdulrahman Muhammad Alahmad, Hamzah Ali Saadoun, Jana Wafek Khaddour, Ahmad Muhammad Zaher Harba, Esraa Gehad Ghazali, Mohammad Wisam Al-Nahas, Zaidan Hazam Zaidan, Tamim Abo Obaid, Ahmad Abdul Hakim Alhamid, Allaith Abo Ghabra, Hebatallah Nasser Fadhl, Badr Mohammed Baras, Afia Tahsin Shobnom, Golam Moktadir, Muhammad Taha Zaman, Saralees Nadarajah, Alhussien Abdelbagi Mohamed Elfaki, Alhasan Abdelbagi Mohamed Elfaki, Mohammed Ali Saghir, Zahin Zeima, Amjad Suliman Almezaal, Reham Almukbel

**Affiliations:** 1 Faculty of Medicine, Al-Sham Private University, Damascus, Syrian Arab Republic; 2 Department of Gastroenterology, Damascus Hospital, Damascus, Syrian Arab Republic; 3 Department of Radiology, Syrian Arab Red Crescent Hospital, Damascus, Syrian Arab Republic; 4 Department of Cardiology, Damascus Hospital, Damascus, Syrian Arab Republic; 5 Department of Nephrology, Syrian Arab Red Crescent Hospital, Damascus, Syrian Arab Republic; 6 Department for Orthopedic Surgery, Syrian Arab Red Crescent Hospital, Damascus, Syrian Arab Republic; 7 Department of General Surgery, Syrian Arab Red Crescent Hospital, Damascus, Syrian Arab Republic; 8 Department of Plastic and Reconstructive Surgery, Syrian Arab Red Crescent Hospital, Damascus, Syrian Arab Republic; 9 Department of Respiratory System Disease, Syrian Arab Red Crescent Hospital, Damascus, Syrian Arab Republic; 10 Department of Internal Medicine Disease, Syrian Arab Red Crescent Hospital, Damascus, Syrian Arab Republic; 11 Department of Neurology, Syrian Arab Red Crescent Hospital, Damascus, Syrian Arab Republic; 12 Department of Orthopaedic surgery, Al-Razi hospital, Aleppo, Syrian Arab Republic; 13 Faculty of Medicine, Lattakia University, Lattakia, Syrian Arab Republic; 14 Medical Education, University of Aden, Aden, Yemen; 15 Department of Science and Technology, Hadhramout University, Mukalla, Yemen; 16 MedEasy Healthcare Limited, Dhaka, Bangladesh; 17 Medical Officer, Ministry of Health, Maldives, Maldives; 18 Coventry University, Coventry, UK; 19 Department of Mathematics, University of Manchester, Manchester, UK; 20 Faculty of Medicine, Al-Neelain University, Khartoum, Sudan; 21 Faculty of Medicine, Cairo University, Cairo, Egypt; 22 Dhaka Medical College, Dhaka, Bangladesh; 23 Department of General Surgery, Damascus Hospital, Damascus, Syrian Arab Republic; 24 Faculty of Medicine, Al-Sham Private University, PhD in Medical Biochemistry and Molecular Biology, Damascus, Syrian Arab Republic

**Keywords:** Conflict setting SSI prevalence, Cross-sectional, Hair removal SSI prevention, Healthcare worker SSI training, LMIC KAP, Surgical site infection, Syria

## Abstract

**Background:**

Surgical site infections (SSIs), infections at or near surgical incisions, represent 20–30% of nosocomial infections globally, with higher prevalence in low- and middle-income countries such as Syria. This study assessed SSI prevalence in two Syrian hospitals alongside a nationwide evaluation of surgical healthcare workers’ knowledge, practices, compliance, and barriers to WHO/CDC SSI prevention guidelines.

**Methods:**

A cross-sectional survey was conducted among 375 healthcare workers in surgical settings across Syria. A structured questionnaire collected data on demographics, educational background, work experience, self-reported practices, knowledge of WHO guidelines, and perceived barriers to implementation. Composite knowledge and practice scores were calculated. Data were analyzed using descriptive statistics, Pearson correlation, and ANOVA.

**Results:**

Adherence to basic preventive practices was high, including hand preparation (89.33%) and intraoperative sterilization (91.47%). However, gaps persisted in avoiding preoperative shaving, appropriate antibiotic prophylaxis timing and duration, and postoperative antibiotic discontinuation. Major barriers included lack of role models (68%), inadequate training (63%), and staff shortages. Pearson analysis revealed positive correlations between compliance and practice scores (*r* = 0.5203, *p* < 0.001) and compliance and knowledge scores (*r* = 0.3372, *p* < 0.001). Crucially, the weakest correlation was found between knowledge and practice scores (*r* = 0.2662, *p* < 0.001), highlighting a prominent know-do gap. Hospital-reported SSI prevalence was 9.5% in one hospital and 1.47% in the other.

**Conclusion:**

This study identified suboptimal knowledge and inconsistent implementation of high-impact SSI prevention practices among Syrian surgical healthcare workers despite strong adherence to basic aseptic measures. Targeted training, improved surveillance systems, and institutional support are needed to strengthen guideline adherence and reduce preventable SSIs.

## Introduction

Surgical Site Infections (SSIs) are among the most common healthcare-associated infections worldwide and represent a significant cause of postoperative morbidity, prolonged hospital stay, increased healthcare costs, and mortality. SSIs are considered infections that occur at the surgical incision or its surroundings during the first 30 days following the surgical operation, or up to 12 months after surgery if any medical devices are implanted during the procedure [1, 2]. Overall, worldwide, SSIs are estimated to be responsible for about 20% to 30% of all infections associated with healthcare institutions and are regarded as the most common cause of hospital-acquired infections in surgical patients [3, 4]. Moreover, it should be noted that the rate of infections among surgical procedures is significantly higher in low- and middle-income countries compared to the rest of the world, which is three times more prevalent [5]. In the Eastern Mediterranean Region, SSI prevalence is assessed at approximately 7–10% [6]. Surgical site infections can commonly be divided into superficial, deep, and organ/space infections. The type of SSI depends on how deep the infection’s which parts of the body are involved. This classification of SSIs is helpful for doctors in clinical assessment and management, as deeper infections are typically associated with more serious outcomes [7]. Risk factors for developing SSIs can be divided into patient-related, operative, and hospital factors. Such factors like comorbidities, the period of surgery, and hospital practices and infection prevention are associated with increased risks [8]. All these things play a role in determining the likelihood of infection after surgery [9]. Physicians’ knowledge and adherence to evidence-based guidelines play a crucial role in preventing SSIs. To minimize the infection rate, it is important to identify potential risk factors, use antibiotics for prophylaxis, and perform asepsis in the operation process [10, 11]. Nevertheless, previous studies have revealed that the knowledge of the infection risks and practice of surgeons is inadequate despite performing their operations in hospitals [12, 13]. In the case of conflict-affected countries like Syria, poor functioning of the healthcare system, lack of medical supplies, overcrowded wards, and absence of continuous medical education leads to a higher rate of SSIs. Despite these facts, there is a shortage of evidence about SSI prevalence and surgeons’ knowledge in Syria. Hence, this study consists of two separate components: (1) a quantitative evaluation of SSI prevalence among postoperative patients at two tertiary hospitals, and (2) a nationwide cross-sectional survey assessing physicians’ knowledge, attitudes, and practices (KAP) about SSI prevention. This study aimed to determine the prevalence of SSIs and evaluate physicians’ knowledge and barriers regarding SSI prevention guidelines in Syria. The objective of the prevalence assessment was to provide a clinical framework for the impact of SSI in surgical settings. The survey component aimed to evaluate the readiness of healthcare personnel and pinpoint gaps in adherence to the guidelines.

## Materials and methods

### Study design and participants

This study consisted of two complementary cross-sectional components. The first component assessed the prevalence of surgical site infections (SSIs) among operated patients in two tertiary hospitals in Syria. The second component was a nationwide questionnaire-based survey of surgical healthcare workers to assess knowledge, practices, perceived barriers, and training needs regarding SSI prevention, utilizing a structured questionnaire based on WHO SSI prevention guidance. The study spanned 4 months, from September 1, 2025, to December 31, 2025. The hospital‑based component was carried out at the Syrian Arab Red Crescent Hospital in Damascus and Latakia Hospital in Latakia, while the survey component included surgeons from multiple public and private hospitals nationwide. Thus, the research was divided into two phases:

Part 1—Hospital-based SSI assessment: This study was conducted at the Syrian Arab Red Crescent Hospital in Damascus and Latakia Hospital in Latakia, Syria. A research team was established due to the challenging environment in the healthcare setting in Syria. The medical records of patients who underwent surgical procedures between February and August 2025 were retrospectively reviewed.

#### Inclusion criteria

- Patients who had undergone major or minor surgical procedures in different specializations, like orthopedic, urology, general surgery, cardiothoracic, neurosurgery, and plastic surgery.
- Patients who were in the hospital for more than 24 h.
- All patients, regardless of age and sex.

#### Exclusion criteria

- Patients discharged within 24 h.
- Patients with incomplete records.
- Patients who developed SSI only after discharge and could not be assessed during the hospital stay.

Surgical site infections (SSIs) were defined and classified in accordance with the World Health Organization (WHO) Global Guidelines for the Prevention of Surgical Site Infection. A total of 284 patients with 27 SSI cases were found who fulfilled the inclusion criteria in the first hospital, and 408 patients with 6 SSI cases in the second hospital. Data were collected directly by the research team from the hospitals. SSIs were identified during the index admission according to predefined clinical criteria, and the primary outcome was the prevalence of SSI among operated patients.

Part 2—Questionnaire‑based survey of healthcare workers: The second part of the study comprised a nationwide survey of healthcare workers involved in perioperative care. A digital, self‑administered questionnaire link was distributed to 375 participants using professional networks, hospital mailing lists, and messaging platforms, including surgeons from diverse specialties in Syria, encompassing both consultants and residents across various public and private hospitals in Syria. Physicians from the internal medicine department were excluded from the questionnaire. A total of 375 healthcare workers completed the questionnaire and were included in the analysis. The sampling strategy was non‑probability (convenience) sampling, which is commonly used for cross‑sectional surveys in constrained settings. Face validity was assessed through expert review, and a pilot test was conducted on 13 healthcare workers. Minor revisions were made based on participant comments to increase questionnaire clarity.

## Sample size

In the hospital-based component, all eligible operated patients during the study period were included; thus, no formal sample size calculation was necessary, as this component relied on the consecutive inclusion of available cases. For the questionnaire-based survey, the target sample size was estimated a priori using a single-proportion formula, predicated on an expected proportion of sufficient SSI prevention knowledge, with a 95% confidence level and a 5% margin of error. To accommodate non-responses, the target sample size was increased by 10–20%. Invitations were sent to approximately 400 eligible participants to ensure adequate final power.

## Tools/instruments

For data collection, the researchers used a structured, self-administered questionnaire developed based on WHO’s guidance for prevention of SSI [14, 15]. It included four sections: (1) Demographic characteristics; (2) Practice section consisting of 10 self-reported items rated on a 3-point Likert scale (Always, Sometimes, Never); (3) Knowledge section comprising 20 multiple-choice questions; and (4) Perceived barriers section consisting of 11 self-reported items rated on a 3-point agreement scale (Agree, Undecided, Disagree).

### Statistical analysis

The data were analyzed using R Software Package. Categorical variables were coded into factors. Three composite scores were formed for statistical analysis:

Practice score—Responses of “always,” “sometimes,” and “never” were scored as 2, 1, and 0 points, respectively, and totaled up (maximum score = 20) over ten questions.

Knowledge score—For scoring, one point was given for each correctly answered multiple-choice question item out of twenty (maximum score = 20) according to the answer key consistent with WHO guidelines.

Barrier score—In order to measure the burden of barriers, responses of “agree,” “undecided,” and “disagree” were coded as 2, 1, and 0 points, respectively, and totaled up (maximum score = 22) over eleven items. Descriptive statistics were calculated for the data. Correlation analyses for determining associations between scores and demographics were run using the Pearson correlation coefficient. An independent samples t-test was used to compare mean scores between trained and untrained participants. One-way Analysis of Variance (ANOVA) was used to compare means within different groups, followed by post hoc comparisons using Tukey’s Honest Significant Difference (HSD) Test. The level of significance set at *P* < 0.05.

### Ethical approval

The research followed the ethical standards outlined by the HelsinkiDeclaration. It received ethical approval from the Research Ethics Committeeat the Syrian Arab Red Crescent Hospital. Permission from the hospitaladministration was sought and obtained before the study began. Priorinformed consent from all participants and confidentiality of their personalinformation were assured.

## Results

### Distribution of demographic characteristics of the studied sample

The majority of the sample consisted of males (57.07%) and females (42.93%). The age distribution of the studied sample revealed that the majority were young adults in the age group of 20-29 years (65.33%), followed by 30-39 years (25.07%), and lastly, aged 40 years or older represented only 9.60%. This implies that there is a young healthcare worker population in surgical wards. Work experience in surgical wards showed that the most frequent category was 1 year or younger with 41.60%, followed by 1–4 years (32.53%). The total proportion with 1–4 years or younger was 74.13%, implying that many healthcare workers did not have sufficient experience in surgical wards. For instance, it can influence the implementation of infection control practices. In terms of education, the most prevalent qualification was MBBS (Bachelor) with 32.53%, followed by board specialists (28.27%), master/MD-MS (18.93%), diploma (18.13%), and consultants (2.13%). However, regarding previous SSI prevention training, 56.53% of the sample population did not undergo any form of training before, while the remaining 43.47% underwent training. Therefore, many workers did not have proper knowledge on the subject matter since less than half had undergone previous training. This implies an opportunity for interventions since trained healthcare workers are always better performing compared to untrained workers, as shown in [Table 1].

**Table 1:** Demographic and professional characteristics of participating healthcare workers (*N* = 375)

Items	Category	No.	%
Sex	Male	214	57.07
	Female	161	42.93
Age (years)	20–29	245	65.33
	30–39	94	25.07
	40–49	24	6.40
	50+	12	3.20
Work experience in surgical ward	Less than 1 year	156	41.60
	1–4 years	122	32.53
	5–9 years	65	17.33
	10 years or more	32	8.53
Educational level	MBBS (bachelor)	122	32.53
	Board (specialist)	106	28.27
	Master/MD-MS	71	18.93
	Diploma	68	18.13
	Consultant	8	2.13
Attended SSI Training Previously	Yes	163	43.47
	No	212	56.53

No bolding inside cells. Title above table. Abbreviations: MBBS, Bachelor of medicine, Bachelor of Surgery; MD , Doctor of medicine; MS, Master of surgery; SSI , Surgical site infection

### Health workers’ compliance with SSI prevention guidelines

In terms of pre-operative practices, surgical hand preparation displayed high adherence, as evidenced by 89.33% of workers who said that they always do this important step in surgery, while none of them never follows this rule. In addition, compliance with the use of chlorhexidine-alcohol antiseptic is also relatively high (66.67% always). Appropriate usage of antibiotics can be noted in 60.27% of people. Nevertheless, significant discrepancies in the preoperative practice could be noted concerning patient bathing/showering: only 42.93% of people always adhere to this rule. The most problematic practice in the pre-op phase includes not shaving patients, as only 21.33% of employees always follow this advice, while 33.33% never do this; 45.33% often violate the practice. Concerning intraoperative practices, it should be admitted that their level is relatively high, as 91.47% of participants always ensure equipment sterility and keep up asepsis; besides, 57.33% always minimize the number of visitors to the operating room. As far as post-operative procedures are concerned, the results indicate both positive and negative trends: 78.40% always examine wounds and provide appropriate dressing, but only 37.87% never continue antibiotics after surgery; 46.93% of participants do so occasionally, 15.20% never follow the advice [Table 2].

### Personal practice of SSI prevention measures

The findings reveal generally strong adherence to several fundamental practices: hand washing before surgical glove use (86.13% always), use of aseptic technique during dressing (84.80% always), use of sterilized dressing material (84.00% always), and assessment and monitoring of surgical sites (85.87% always). Hand washing before and after wound dressing also showed good compliance at 80.80%. The following were some of the improper practices identified. Firstly, the practice of shaving of skin right before surgery, which is an outdated and potentially dangerous practice, was always done by 34.93% and sometimes by 48.00% of respondents, while 17.07% never engaged in this practice. Secondly, advice on washing using antiseptic solutions prior to surgery was rarely followed since only 36.27% always advised on this issue while 28.00% never gave this advice. Thirdly, administering pre-surgery antibiotics after the recommended hour was a consistent problem since only 53.87% of the respondents always did this practice. Fourthly, proper separation of infected and non-infected dressings was only done by 58.93% who always did so and 30.40% never did so. Lastly, wearing a face mask during dressing was always done by 59.73% of the respondents, while 35.73% sometimes did this practice. These findings in [Table 2] highlight specific behavioral targets for educational interventions

**Table 2:** Healthcare workers’ compliance with SSI prevention guidelines and self-reported personal practices (*N* = 375)

Compliance item	Never No. (%)	Sometimes No. (%)	Always No. (%)
*Before surgery*			
Ensure patients bath or shower	57 (15.20)	157 (41.87)	161 (42.93)
Do not shave patients	125 (33.33)	170 (45.33)	80 (21.33)
Only use antibiotics when recommended	37 (9.87)	112 (29.87)	226 (60.27)
Use chlorhexidine-alcohol antiseptic	45 (12.00)	80 (21.33)	250 (66.67)
Surgical hand preparation	0 (0.00)	40 (10.67)	335 (89.33)
*During surgery*			
Limit number of people and door openings	29 (7.73)	131 (34.93)	215 (57.33)
Ensure equipment sterile, maintain asepsis	0 (0.00)	32 (8.53)	343 (91.47)
*After surgery*			
Do not continue antibiotics postoperatively	57 (15.20)	176 (46.93)	142 (37.87)
Check wounds and use standard dressings	9 (2.40)	72 (19.20)	294 (78.40)
*Personal practices*			
Hand wash before and after wound dressing	20 (5.33)	52 (13.87)	303 (80.80)
Hand wash before surgical glove	10 (2.67)	42 (11.20)	323 (86.13)
Pre-op shaving right before surgery	64 (17.07)	180 (48.00)	131 (34.93)
Pre-op antibiotic within 1 h	36 (9.60)	137 (36.53)	202 (53.87)
Advise preop showering with antimicrobial	105 (28.00)	134 (35.73)	136 (36.27)
Use sterilized dressing material	11 (2.93)	49 (13.07)	315 (84.00)
Use aseptic technique during dressing	9 (2.40)	48 (12.80)	318 (84.80)
Assess and monitor surgical site	5 (1.33)	48 (12.80)	322 (85.87)
Separate infected from non-infected dressing	114 (30.40)	40 (10.67)	221 (58.93)
Use face mask during dressing	17 (4.53)	134 (35.73)	224 (59.73)

SSI, Surgical site infection

### Barriers to compliance with SSI prevention guidelines

The most frequently cited barrier was “lack of a professional role model,” with 68.00% of participants agreeing that this impedes compliance. This highlighted the importance of leadership and mentoring in setting and maintaining infection control measures. The third most prevalent barrier was “lack of training on SSI prevention,” which was rated by 62.93% of the respondents, consistent with the previous results showing that over half of the respondents have not undergone any formal training on SSI prevention. The item “unsuitable health worker-patient ratio” was pointed out by 62.13% of the respondents as a barrier, highlighting the systemic problems regarding the work pressure of health care professionals, which may require them to cut corners when it comes to infection control measures. “Lack of knowledge about disinfection” was raised by 58.67% of the respondents, while “lack of evidence-based recommendations” was mentioned by 57.07%, indicating knowledge and resource deficiencies. “Lack of supervision for the infection control committee” was indicated by 54.67% of the respondents, hinting at possible inadequacies in the supervision process. “Lack of supplies for surgery” was listed by 51.20% of the respondents as a potential barrier, showing a resource issue that cannot be fixed with educational interventions alone. Interestingly, only 40.00% of the respondents agreed that “health workers lack required skills. The perception that “some measures are not HCW responsibility” was endorsed by 52.53%, indicating diffusion of responsibility that requires clarification of roles and accountabilities. Intervention required in this context can be expected on individual, organizational, and systemic levels, because barriers to change exist on all these levels. They relate to knowledge deficits, lack of resources, insufficient supervision, and excessive workload [Table 3].

**Table 3:** Barriers to compliance with ssi prevention guidelines (*N* = 375)

Barriers	Disagree No. (%)	Undecided No. (%)	Agree No. (%)
1. Inadequate supply of surgical consumables	82 (21.87)	101 (26.93)	192 (51.20)
2. Lack of supervision of infection control committee	100 (26.67)	70 (18.67)	205 (54.67)
3. Inadequate knowledge about disinfection	92 (24.53)	63 (16.80)	220 (58.67)
4. Lack of training on SSI prevention	80 (21.33)	59 (15.73)	236 (62.93)
5. Lack of evidence-based recommendations	75 (20.00)	86 (22.93)	214 (57.07)
6. Unsuitable health worker-patient ratio	64 (17.07)	78 (20.80)	233 (62.13)
7. Lack of professional role model	43 (11.47)	77 (20.53)	255 (68.00)
8. Poor integration of research findings	97 (25.87)	100 (26.67)	178 (47.47)
9. Health workers do not have enough time	103 (27.47)	93 (24.80)	179 (47.73)
10. Health workers lack required skills	115 (30.67)	110 (29.33)	150 (40.00)
11. Some measures not HCW responsibility	97 (25.87)	81 (21.60)	197 (52.53)

Table 3 presents healthcare workers' perceptions of barriers hindering compliance with SSI preventionguidelines, with responses categorized as disagree, undecided, or agree

### Knowledge of health workers regarding SSI prevention guidelines

Significant differences were seen in the levels of knowledge about different practices. Most health workers knew how to prepare their hands surgically before operation (88.27%), how to manage the circulating volume (87.20%) adequately, and how to prepare patients for surgery in terms of bathing (85.87%). The knowledge concerning the nutrition of patients was also quite high—it made up 80.00%. But there were also certain gaps in knowledge. The most concerning knowledge gap is to hair removal; only 35.47% of respondents are aware that it is not recommended and that clippers, not razors, should be utilized when necessary. Such data corresponds well with the findings provided in Table 2. In addition to the poor compliance with guidelines found in Table 3, we should expect such knowledge gaps to be addressed. Only 57.87% of health workers knew how to determine the optimal time of antibiotic prophylaxis. Moreover, 42.67% of them did not understand how to perform surgical preparation of the patient’s surgical site in case alcohol-based chlorhexidine gluconate (CHG) was used. Oxygenation management had poor knowledge scores (42.13%), while immunosuppressive agent administration was understood by 30.67% only. Finally, there was controversy concerning the duration of post-surgical prophylaxis: 50.40% understood correctly that prophylaxis should not be extended more than 24 h.

### Compliance score levels

The average compliance score obtained is 18.61 (SD = 4.22), which shows moderate compliance. Dividing this score into three compliance score levels shows that the highest percentage is represented by moderate compliance 154 (41.07%, scores 16–21). The next category, low compliance 148 (39.47%, scores 9–15) is represented by slightly fewer workers, while only 73 (19.47%) achieve high compliance (scores 22–27). The data show that nearly four in every ten health care professionals have low compliance, which represents a serious patient safety issue. High compliance is achieved by less than one-fifth of workers, which suggests an opportunity for improvement in compliance practices. The average compliance score of 18.61 represents about 69% of the maximum possible score, meaning that healthcare workers adhere to almost two-thirds of the best practices. This, along with low compliance, shows that there is an urgent need to implement interventions to increase the number of practices in place for preventing SSIs. A high standard deviation of 4.22 means that compliance scores differ greatly from person to person, which suggests that some healthcare workers perform much better and that mentoring could be an effective strategy. The average compliance score was 18.61 ± 4.22, with the majority of participants (41.07%) falling into the moderate compliance category, as shown in Table 4.

**Table 4:** Compliance score levels (*N* = 375)

Score level	No.	%
Mean +/− SD = 18.61 +/−4.22		
Low (9–15)	148	39.47
Moderate (16–21)	154	41.07
High (22–27)	73	19.47

N, number of participants; %, percentage; SD, Standard deviation. The overall mean compliance score was 18.61 ± 4.22 out of a maximum possible score of 27. Categorical data are presented as counts and percentages, while the global score is presented as Mean ± SD

### Knowledge score levels

On average, the knowledge score is 10.94 (SD = 4.88), which means that healthcare workers answered about 55% of the questions correctly. If divided into three categories, we can see that the highest percentage is made up of healthcare workers with moderate knowledge of SSI guidelines, 158 (42.13%, scores 7–13). Low-level knowledge of SSI prevention guidelines is demonstrated by 126 (33.60%) of healthcare workers (scores 0–6), whereas high knowledge is shown by only 91 (24.27%). One-third of workers show extremely low knowledge of SSI guidelines. Insufficient knowledge contributes to low compliance rates. Only slightly more than one-quarter of workers know much about how to prevent SSIs, which means that most health care workers lack sufficient knowledge. SD of 4.88 suggests great variations in knowledge of different workers, which means that there may be workers with perfect knowledge and those who know practically nothing. Knowledge levels regarding SSI guidelines were categorized, revealing that 42.13% of healthcare workers had moderate knowledge, while only 24.27% demonstrated high knowledge [Table 5]

**Table 5:** Knowledge score levels (*N* = 375)

Score level	No.	%
Mean +/− SD = 10.94 +/− 4.88		
Low (0–6)	126	33.60
Moderate (7–13)	158	42.13
High (14–20)	91	24.27

N, number of participants; %, percentage; SD, Standard deviation. The overall mean knowledge score was 10.94 ± 4.88 out of a maximum possible score of 20. Categorical data are presented as counts and percentages, while the global score is presented as Mean ± SD

### Practice score levels

Mean practice score was 22.94 (SD = 5.37), which is equivalent to 76% of the attainable maximum score. It is evident that this number is way above mean compliance score (69%) and mean knowledge score (55%). It suggests that self-reported practice seems to be way ahead of both compliance level and knowledge about SSI prevention measures. Categorizing participants according to these three measures, we see that the largest category of people (43.20%) is in the moderate practice range (scores 17–23). There are fewer people who have high-level practices (33.07% of participants had scores 24–30). At the same time, 23.73% of participants fall into the low practice category (10–16). Importantly, one-third of all participants reported having good practices, whereas 19.47% have high compliance scores and 24.27% report high knowledge levels. Such discrepancy among the three categories requires us to consider various explanations, such as social desirability bias in practice self-reporting or real differences between knowledge, compliance, and practice. As well as the fact that practice is influenced by some other factors apart from knowledge of the person. Relatively large mean score suggests that many recommended practices are already incorporated into routine behaviors. However, the fact that 23.73% still show low practice scores calls for certain interventions among these people. Self-reported practice scores were higher than knowledge scores, with a mean of 22.94 ± 5.37. The distribution of these practice levels is detailed in Table 6.

**Table 6:** Practice score levels (*N* = 375)

Score level	No.	%
Mean +/− SD = 22.94 +/− 5.37		
Low (10–16)	89	23.73
Moderate (17–23)	162	43.20
High (24–30)	124	33.07

N, number of participants; %, percentage; SD, Standard Deviation. The overall mean practice score was 22.94 ± 5.37 out of a maximum possible score of 30. Categorical data are presented as counts and percentages, while the global score is presented as Mean ± SD

### Association between training and outcomes

As shown by the findings presented above, training is associated with improved outcomes in each measure (knowledge, compliance, and practice). For instance, mean compliance scores were 19.84 (SD = 4.05) in the trained group and 17.67 (SD = 4.18) in the untrained one; thus, there is a statistically significant difference (t = 4.73, *p* < 0.001). Therefore, training is associated with significantly higher compliance with SSI prevention guidelines. Mean knowledge score was 12.15 (SD = 4.52) in the trained group and 9.97 (SD = 4.98) in the untrained one (t = 3.64, *p* < 0.001), showing that training improves knowledge. Most importantly, training is significantly associated with higher practice score. Participants in the trained group obtained 24.21 (SD = 4.89) scores against 21.50 (SD = 5.42) among the untrained (t = 4.31, *p* < 0.001). The difference between the two groups was largest regarding practice score, which means that training not only improves people’s knowledge of SSI prevention but also influences people’s practices. The findings suggest that training needs to be expanded and reinforced.

### Logistic regression–factors associated with training attendance

The model was statistically significant (χ^2^ = 42.18, *p* < 0.001) with R2 of 0.18, suggesting that the predictors explain 18% of the variability in training attendance. Only one characteristic among demographic variables reached statistical significance, namely work experience. Workers with five or more years of experience had 1.72 times higher odds of being trained in SSI compared to workers with less than five years of experience (adjusted OR = 1.72; 95%CI: 1.10–2.71; *p* = 0.017). It can be concluded that more experienced workers either participated in more SSI trainings throughout their careers, or such trainings preferred older employees. The other demographic factors were not statistically significantly associated with training attendance. Importantly, all three outcome measures were strongly associated with training attendance. Participants who scored highly on the compliance scale had 2.91 times higher odds of attending training compared to those with low compliance score (adjusted OR = 2.91; 95%CI: 1.69–5.03; *p* < 0.001). Moreover, those who scored highly in the knowledge section had 2.03 times higher odds of attending SSI training (adjusted OR = 2.03; 95%CI: 1.21–3.41; *p* = 0.008). Participants who scored highly in the practice section were 2.41 times more likely to attend SSI training (adjusted OR = 2.41; 95%CI: 1.46–3.99; *p* = 0.001). These statistically significant results support the t-test analysis’s findings and provide further evidence that training attendance is related to enhanced compliance, knowledge, and practice scores. However, the direction of this relationship cannot be determined because of the study’s cross-sectional design. Healthcare professionals who are more interested or have a higher level of expertise may also be more inclined to participate in training programs, which may lead to improved results. However, the results demonstrate the potential benefits of SSI prevention training and encourage its wider use [Table 7].

**Table 7:** Logistic regression–factors associated with training attendance

Predictor	Comparison	Crude OR	Adjusted OR	*p*-value
Work experience	5 + years vs. < 5	1.67 (1.09–2.57)*	1.72 (1.10–2.71)*	0.017*
Education	Board or higher	1.34 (0.89–2.03)	1.29 (0.84–1.99)	0.25
Compliance score	High vs. other	2.84 (1.68–4.81)***	2.91 (1.69–5.03)***	< 0.001***
Knowledge score	High vs. other	2.17 (1.33–3.55)**	2.03 (1.21–3.41)**	0.008**
Practice score	High vs. other	2.53 (1.55–4.12)***	2.41 (1.46–3.99)***	0.001***

OR, Odds ratio; CI, Confidence interval. Adjusted ORs were derived from multivariable logistic regression analysis. *p* < 0.05; ** *p* < 0.01; *** *p* < 0.001

### Logistic regression analysis of compliance, practice, and knowledge level associated with demographic factors

It is noted that the model is statistically significant (LR χ^2^ = 26.39, *p* = 0.0094), meaning that the predictors chosen can explain the variance in compliance. As for the demographic variables, sex was identified as a significant predictor, where males have significantly greater odds than females to be classified in a higher compliance group (coefficient = 0.4685, *p* = 0.045). The implication is that males tend to adhere more to the SSI preventive procedures, an interesting finding which calls for further research into its causes. In addition, experience proved to negatively impact compliance where those who had 10 + years of experience were significantly less likely to be in the higher compliance group than those having between 1 and 4 years of experience (coefficient = − 2.7864, *p* = 0.043). It might mean that with time, the experienced employees get used to working under certain conditions which make them less compliant with new recommendations. On the contrary, those who started working recently might be more aware of the current issues related to SSI prevention because of having attended training courses more recently. Age, education level (Board/Specialist, Consultant, Diploma, Master/MD), and training attendance did not prove their significance in relation to the compliance level (*p* > 0.05).

### Ordered logistic regression: demographic factors associated with knowledge level

It should be stated that the model proved to be a great fit for the analysis and had high significance (LR χ^2^ = 38.94, *p* = 0.0001). Age turned out to be the only factor which proved to have statistically significant effect on respondents’ ability to acquire knowledge on SSI prevention (the odds of those aged 40–49 being classified as having higher knowledge level were lower than the odds of those aged 20–29 by 2.6770 (*p* = 0.014)). It can be concluded that middle-aged health workers are likely to be aware of fewer prevention recommendations than their peers due to updates in SSI prevention guidelines provided during more recent trainings, whereas middle-aged workers have failed to benefit from further continuing education. There were two categories in regard to educational background that were close to being statistically significant: those with Board/Specialist qualification possessed knowledge of SSI prevention to some extent higher (*p* = 0.064; coefficient = 0.6099) than those with MBBS qualification and those who held a diploma (coefficient = 0.5983; *p* = 0.055). It might be supposed that having obtained additional education, workers tend to develop more profound understanding of preventive actions, but still these tendencies do not prove to be statistically significant. Sex, age, experience, Consultant qualification, Master/MD degree, and training attendance turned out to be irrelevant predictors (all *p* > 0.05). According to pseudo R² equaling 0.0564, the proportion of variance explained by demographic factors can be considered rather low.

### Ordered logistic regression: demographic factors associated with practice level

Contrary to previous models, this model was not statistically significant (LR χ^2^ = 14.33, *p* = 0.2804), suggesting that the predictors used do not account for variations in practice levels. Even though the whole model was not statistically significant, experience became a statistically significant predictor, with individuals having at least ten years of experience showing a relatively high probability of being associated with lower practice levels than those with one to four years of experience (coefficient = − 3.5281, *p* = 0.016). Consistent with the compliance model, this finding supports the idea that experienced practitioners could develop undesirable behavior or resistance to adopting evidence-based practices, whereas younger practitioners tend to have a higher adherence to practice guidelines. No other predictors were found to be statistically significant (sex, all age categories, all education categories [Board/Specialist, Consultant, Diploma, Master/MD], and training attendance). Since the model was statistically insignificant (*p* = 0.2804) and had a very low pseudo R^2^ value of 0.0341, it can be inferred that the demographic and professional factors analyzed here are poor predictors of practice behaviors [Table 8].

**Table 8:** Ordered logistic regression: significant demographic predictors

Outcome	Predictor	OR	95% CI	*p*-value
Compliance level (low→high)	Male vs. female	1.60	1.01–2.53	0.045
	10 + years vs. 1–4 years of experience	0.06	0.004–0.91	0.043
Knowledge level (low→high)	Age 40–49 vs. 20–29	0.07	0.008–0.58	0.014
Practice level (low→high)	10 + years vs. 1–4 years of experience	0.03	0.002–0.52	0.016

OR, Odds ratio; CI, Confidence interval. An OR > 1 indicates higher odds of being in a higher outcome category, while an OR < 1 indicates lower odds

### Correlation matrix: compliance, knowledge, and practice scores

All correlations between the variables were positive and statistically significant at *p* < 0.001, which means that as the values on one scale increased, there was an increase in the other scores as well. The highest correlation existed between compliance and practice scales (*r* = 0.5203). As can be seen from this result, there is a moderate relationship between these two scales; in other words, there is a positive correlation that indicates that the people who are compliant in their practice are also likely to be compliant with institutional regulations. It is noteworthy that the existence of this correlation is positive since it suggests congruence between reported behavior and guideline adherence. However, a weaker but still moderate relationship existed between compliance and knowledge (*r* = 0.3372). In other words, as the knowledge level of healthcare professionals increased, they became more compliant. However, it is important to admit that the correlation was weak and did not suggest any strong relation. Most importantly, the lowest correlation between the selected scales was found between knowledge and practice (*r* = 0.2662). The existence of such a low correlation means that a significant know-do gap exists, and the increase in knowledge does not contribute significantly to practice [Table 9].

**Table 9:** Correlation matrix: compliance, knowledge, and practice scores

Variable	Compliance score	Knowledge score	Practice score
Compliance score	1.0000		
Knowledge score	0.3372*	1.0000	
Practice score	0.5203*	0.2662*	1.0000

*p < 0.001. r = Pearson correlation coefficient*

## Discussion

Surgical site infections (SSIs) are defined as infections related to an operative procedure that occur at or near the surgical incision site [16, 17]. They are the most common healthcare infections worldwide. Additionally, SSIs are associated with serious complications such as increased morbidity and prolonged hospital stay due to sepsis. Prevention largely depends on adherence to infection control measures by healthcare workers. In the present study, we investigated health workers’ knowledge, compliance, and practices regarding surgical site infection (SSI) prevention in surgical wards and the prevalence of SSI in two tertiary hospitals in Syria. The prevalence of SSI during index hospitalization at the Syrian Arab Red Crescent Hospital was 9.5%. Additionally, a low SSI prevalence of 1.47% was identified in Latakia Hospital. According to reports, SSI rates in low- and middle-income countries (LMICs) range from approximately 4 to 18%, with wider ranges up to 30% depending on the procedure type and monitoring techniques [18, 19]. The prevalence found in our study is within this range. Approximately 7% in Gulf Cooperation Council (GCC) countries [20], 1.5–20% in Europe [21], 9.2–15.8% in Ethiopia [22], 7% in Jordan [23], 7.8–11.8% in Egypt [24], 12% in India, and 38% in Nigeria [25] while some settings in Nigeria and sub-Saharan Africa have reported rates exceeding 14% [26]. Conversely, high-income countries, particularly the United States and the United Kingdom, typically report lower SSI rates [27, 28], which are commonly linked to better infection control practices, more standardized perioperative care, and easier access to resources. Therefore, it is clear that differences in SSI rates across studies and geographic areas may be due to variations in infection control practices, antibiotic prophylactic procedures, preoperative screening measures, and patients’ comorbid conditions [29]. Similarly, variations in study designs, definitions of SSI, as well as duration of follow-up, preclude direct comparison of rates of SSI among various studies; hence, it is important to interpret these results in context while comparing them with previous literature [30]. Healthcare workers showed low levels of knowledge in general, with only a minority showing high knowledge levels despite having better self-reported practices. Significant perceived barriers concerning training, resources, and organizational support were noted. This research reveals an important gap between knowledge and practice among healthcare professionals. Self-reported practice scores were relatively high (76.5%), whereas knowledge scores were markedly lower (54.7%). The gap is further confirmed by a weak correlation coefficient between knowledge and practice (*r* = 0.2662, *p* < 0.001). This suggests that adherence to SSI guidelines may be more influenced by clinical practice than by a comprehensive understanding of the existing guidelines. The importance of attending training sessions was seen to be a significant and modifiable component because those individuals who attended training achieved much better scores in compliance, knowledge, and practice compared to those who did not attend any training. When interpreted in light of international guidelines such as the WHO Global Guidelines and CDC recommendations for SSI prevention, our findings reveal important deviations from best practice, particularly in the areas of preoperative skin preparation, hair removal, and duration of antimicrobial prophylaxis. Prior SSI prevention training attendance was associated with consistently superior performance: compliance, knowledge, and practice scores. A medium to large effect size (Cohen’s d ≈ 0.5) aligns with the results of LMIC-focused training initiatives that show the clinical applicability of interventions. For instance, the Clean Cut initiative in Ethiopia resulted in a 35% relative decrease in the postoperative risk of infections following IPC training among 3,364 patients [31], whereas the task-based training initiative in Pakistan led to significant knowledge-to-action transfer, thus closing the knowledge-implementation gap [32]. The research literature provides ample evidence for consistently favorable outcomes. Specifically, according to studies conducted by Abida Parveen et al. (2025) and Dr. Tariq Aziz et al. (2025) [32, 33], educational interventions, including lectures and practical exercises, significantly increase knowledge test scores, promote adherence to best practices, and boost confidence in infection control techniques. This is particularly effective in hand hygiene as well as sterile technique. In the meta-analyses done by Berhe, it has been proven that there is a reduction of 15–95% in SSI in Sub-Saharan Africa with the use of multimodal training [34], and in the study conducted by Habtie et al. [35], it has been proven that this increases knowledge of SSI in nurses in the study. These consistent findings establish training as a high-leverage, modifiable strategy for Syrian surgical quality improvement; however, longitudinal studies are needed to validate sustained improvements and SSI incidence reductions. Our findings demonstrate generally strong adherence to key SSI prevention practices among Syrian healthcare workers, particularly in fundamental measures such as hand hygiene before glove use (86.13%), aseptic dressing technique (84.80%), and surgical site monitoring (85.87%). The results of this study indicate to a significantly high level of awareness and implementation of fundamental infection prevention concepts. In contrast, research conducted in Ghana [20, 36] found that 43% of healthcare workers had poor SSI preventive strategies and just 57% had good ones. A multicenter RCT involving 13 hospital units in four US academic institutions showed that 41% of patients followed standard care by washing their hands before donning gloves [37]. This indicates that the overall level of adherence observed in our study appears comparatively higher in several essential domains of SSI prevention. Preoperative hair removal is an instance of an ongoing gap between knowledge and practice in a wide range of healthcare settings. Despite WHO recommendations clearly advising against shaving, only 21.3% of healthcare professionals in our Syrian study consistently avoided it, and only 35.5% of them had adequate knowledge. Such an assumption is consistent with the evidence from rich countries and is confirmed by studies conducted in Sudan, where more than half of the surgical personnel erroneously stated their belief in the necessity of shaving before operations. Important inconsistencies were identified regarding both the performance and understanding of the research basis for avoiding shaving among Toronto surgeons [38]. The procedure of shaving before the operation continues to be widely practiced, resisting recommendations, and this phenomenon has roots in the convergence of contexts ranging from conflicts to academia. This finding is consistent in both high-resource (Canada) and low-resource (Syria/Sudan) settings, indicating that behavioral and sociocultural factors other than resource availability are responsible for its persistence. Instead of depending only on general SSI training programs, targeted de-implementation strategies, that address ingrained professional norms are recommended. The observation that practice scores exceeded knowledge and compliance scores suggests a broader know–do gap in which routine behaviours and local norms, rather than formal guideline awareness, drive SSI-related practices—consistent with reports from other LMIC surgical settings [39, 40]. Taken together, these findings support a multi-pronged strategy for SSI prevention in Syria that combines mandatory, recurrent training for all perioperative staff with institutional measures such as standardized checklists, written protocols, and routine audit and feedback, embedded within broader post-conflict health system recovery efforts. Notably, this research offers several important contributions to the existing literature. This is the first study to jointly assess SSI prevalence and SSI prevention knowledge, practices, and barriers among surgical healthcare workers in Syria, providing rare empirical data from a protracted conflict setting. By combining patient-level SSI surveillance with a nationwide survey of 375 perioperative staff, the study offers a multi-level perspective on both clinical outcomes and the underlying behavioural and system-level determinants. Using an instrument based on the WHO guidelines, along with composite scores in addition to multivariate regression modeling, increases the internal validity of the study. The findings emphasize the dire need for well-organized and context-specific prevention programs for SSIs within overall health system recovery strategies, since Syria has been exposed to constant disruption of its health care facilities through conflicts.

### Limitations and recommendations

Some important limitations must be taken into account when analyzing the outcomes from the present study. Firstly, data collection procedures took place directly through research team members. As a result, response and social desirability bias might have emerged, especially regarding the reporting of certain clinical practice-related issues. These biases were reduced by implementing strict data collection procedures consistently across all participants. Secondly, a convenience sampling strategy and a cross-sectional design were used in this study. Data were collected at a single time point, making it impossible to establish causal relationships between the studied variables. Thirdly, the data obtained from hospitals were limited to only two tertiary institutions in Syria. As a consequence, the observed SSI prevalence rates cannot be generalized to other regions in the country. SSI rates may differ substantially across hospitals because of variations in case mix and available financial, organizational, and human resources. The retrospective analysis was performed using data collected from hospitals’ internal information systems, which did not include standardized post-discharge surveillance; therefore, some post-discharge SSI cases may have been underreported or missed. Future studies will require collecting data from a larger number of institutions using both prospective and retrospective designs. It is important to include healthcare facilities from different sectors and geographic regions of Syria (public, private, teaching, military, etc.). This would provide a more accurate national picture of the current situation and allow the evaluation of factors that may influence SSI rates. In addition, observational studies alone cannot establish causal relationships. Therefore, future interventional and longitudinal studies are needed to further evaluate the effectiveness of SSI prevention strategies.

## Conclusion

Overall, we found a notable surgical site infection prevalence alongside suboptimal knowledge, with inconsistent high-impact practices among Syrian healthcare workers, despite good basic asepsis. This matters because it reveals a modifiable knowledge-practice gap driving preventable SSIs in Syria's strained healthcare system. This suggests implementing mandatory multimodal training, local SSI surveillance with post-discharge follow-up, and resource procurement via WHO partnerships to reduce infection rates sustainably.

## Data Availability

No datasets were generated or analysed during the current study.

## Abbreviations

SSI: Surgical site infection
WHO: World health organization
LMIC: Low- and middle-income countries
GCC: Gulf cooperation council
